# Continuous theta burst stimulation to the medial posterior cerebellum impairs reversal learning in healthy volunteers

**DOI:** 10.3758/s13415-025-01273-5

**Published:** 2025-03-26

**Authors:** Eline S. Kruithof, Eva M. Drop, Daan Gerits, Jana Klaus, Dennis J. L. G. Schutter

**Affiliations:** Department of Experimental Psychology, Helmholtz Institute, Utrecht University, Heidelberglaan 1, 3584 CS Utrecht, The Netherlands

**Keywords:** Behavioral adaptation, Cerebellum, Punishment, Reversal learning, Reward, Transcranial magnetic stimulation

## Abstract

The role of the cerebellum in associative learning and context-updating implies involvement in learning reward-punishment contingencies. This study examined the direct contribution of the cerebellum to reward- and punishment-based reversal learning. A total of 111 healthy right-handed adult volunteers received continuous theta burst stimulation (cTBS) to either the medial posterior cerebellum (*n* = 37), right posterolateral cerebellum (*n* = 37), or right occipital lobe (*n* = 37) in this single-blind between-subjects study. A gambling task with two changing reward-punishment contingencies (reversals) was administered to assess reversal learning rate and the implementation of the optimal strategy as primary endpoints. As secondary endpoints, heart rate variability (HRV), state anxiety, state anger, trait aggression, and trait impulsivity were assessed to examine interactions with cerebellar cTBS on the implementation of the optimal strategy. Results showed that medial posterior cerebellar cTBS compared with right posterolateral cerebellar and right occipital lobe cTBS reduced learning rate after the first reversal and diminished the implementation of the optimal strategy after learning the second reversal. No interactions of cTBS with HRV, state anxiety, state anger, trait aggression, and trait impulsivity on the implementation of the optimal strategy were observed. Our findings provide evidence for involvement of the cerebellum in reward- and punishment-based reversal learning and behavioral adaptation.

**Supplementary Information:**

The online version contains supplementary material available at 10.3758/s13415-025-01273-5.

## Introduction

In addition to the well-established role of the cerebellum in vestibular and motor functions (Manto et al., 2012; Stoodley & Schmahmann, 2009; Stoodley et al., 2012), growing evidence shows involvement of the cerebellum in cognitive (Keren-Happuch et al., 2014; Stoodley & Schmahmann, 2009) and affective processes (Adamaszek et al., 2017; Keren-Happuch et al., 2014; Stoodley & Schmahmann, 2009). To accommodate these processes, the cerebellum is argued to form internal prediction models representing learned action-outcome associations (Blakemore et al., 2001; Ebner & Pasalar, 2008; Ito, 2008; Koziol et al., 2014; Wolpert et al., 1995, 1998). These inferential models are based on comparisons between anticipated and actual outcomes as part of a prediction error minimization routine serving to reduce uncertainty and maximize performance (Friston, 2010; Shadmehr et al., 2010; Wolpert et al., 1995). Reward- and punishment-related feedback signals provide critical input to the internal model as qualitative indices of the prediction error minimization routine (Kruithof et al., 2023).

An unexpected punishment or lower than anticipated reward following an action requires updating of the internal model to enable correct future reward-punishment outcome predictions of actions. However, the need to update the internal model is context-dependent, such that updating is required only when (novel) action-outcome associations turn out to be systematic (i.e., non-random) in a given situation. The ability to learn action-outcome associations across contexts with different reward-punishment contingencies is called reversal learning and deemed critical for mental flexibility and behavioral adaption (Cools et al., 2002; Izquierdo et al., 2017).

Experimental brain research in animals, as well as studies in patients with cerebellar lesions and healthy volunteers have found evidence for involvement of the posterior cerebellum in reward- and punishment-related motivational processes (Berlijn et al., 2024; Kruithof et al., 2023) and reversal learning (Badura et al., 2018; Dickson et al., 2010, 2017; Kelly et al., 2020; Peterburs et al., 2018; Stoodley et al., 2017; Thoma et al., 2008; Tsai et al., 2012; Verpeut et al., 2023). Moreover, cerebellar lateralization of motivational direction has recently been proposed, which postulates that relative left-sided cerebellar activity is associated with punishment- and avoidance-related behavior whereas relative right-sided cerebellar activity is associated with reward- and approach-related behavior (Kruithof et al., 2022, 2024, 2025; Schutter, 2020), extending the well-documented frontal cortical lateralization of motivational direction (Kelley et al., 2017).

The aim of the current study was to examine the direct contribution of the cerebellum to reward- and punishment-based reversal learning. To this end, continuous theta burst stimulation (cTBS) was applied to transiently perturb the medial and right lateral posterior cerebellum, and the right occipital lobe as an active control site in healthy adult volunteers. Continuous theta burst stimulation is a repetitive transcranial magnetic stimulation (rTMS) protocol, which consists of three-pulse high-frequency bursts that are repeated every 200 ms for 40 s in total and decreases cortical excitability (Huang et al., 2005; Wischnewski & Schutter, 2015). Available evidence suggests that the effect of cerebellar cTBS can last up to 30 min (Popa et al., 2010; Strzalkowski et al., 2019). In addition, the cTBS protocol was chosen for this study owing to its shorter application time compared with traditional rTMS protocols. The contribution of the right posterolateral hemisphere was investigated based on the cerebellar lateralization of motivational direction that attributes a role of the right posterolateral cerebellum to reward-related motivational processes (Kruithof et al., 2022, 2024; Schutter, 2020). Reward- and punishment-based reversal learning is assumed to involve a more cognitive component, which relates to the ability to make a causal connection between actions and consequences, and a more affective component pertaining to the sensitivity to reward and punishment feedback signals. As primary endpoints, we hypothesized that the former would be impaired following cTBS to the right posterolateral cerebellum given its role in higher-order cognitive processes (Keren-Happuch et al., 2014; King et al., 2019; Stoodley & Schmahmann, 2009), whereas the latter would be impaired following cTBS to the medial posterior cerebellum owing to its role in affective processes (Baumann & Mattingley, 2012; Stoodley & Schmahmann, 2009). Furthermore, as secondary endpoints, two additional aspects were explored. First, because mental flexibility is positively associated with heart rate variability (HRV; Hovland et al., 2012; Howell et al., 2022), we examined potential interactions between HRV and cerebellar cTBS on reversal learning. Second, psychological research has shown that reversal learning and the effects of non-invasive brain stimulation can be influenced by affective states and traits (Franken et al., 2008; Mitchell et al., 2006; Rosa-Alcázar et al., 2020; Schutter et al., 2023; Xia et al., 2021). To that end, we investigated possible interactions of self-reported state anger, state anxiety, trait aggression, and trait impulsivity with cerebellar cTBS on the affective component of reversal learning.

## Methods

### Participants

One hundred and eleven healthy, right-handed, non-smoking adult volunteers (18 males, age: *M* = 22.51, *SD* = 2.74, range = 18–35) participated in the study in exchange for monetary compensation. Participants were excluded if they had a history of neurological or psychiatric conditions, family history of epilepsy, heart disease, metal in the head, pacemaker or neurostimulator, electronic hearing aid, head trauma or underwent brain surgery, were pregnant, or used drugs or medication (except for oral contraceptives). The required sample size was estimated by performing a power analysis in G*Power 3.1 (Faul et al., 2007). An effect size of *f* = 0.3 was used, based on a meta-analysis that examined the effects of cerebellar transcranial magnetic stimulation (TMS) on accuracy in cognitive tasks (Gatti et al., 2021). A one-way ANOVA with this effect size, an α of 0.05, and power of 0.8 with three groups resulted in a required sample size of 111 participants. All participants gave written informed consent. The study was approved by the medical ethics committee NedMec (protocol number NL82216.041.22) and was performed in accordance with the Declaration of Helsinki.

### Reversal learning gambling task

**Fig. 1:**
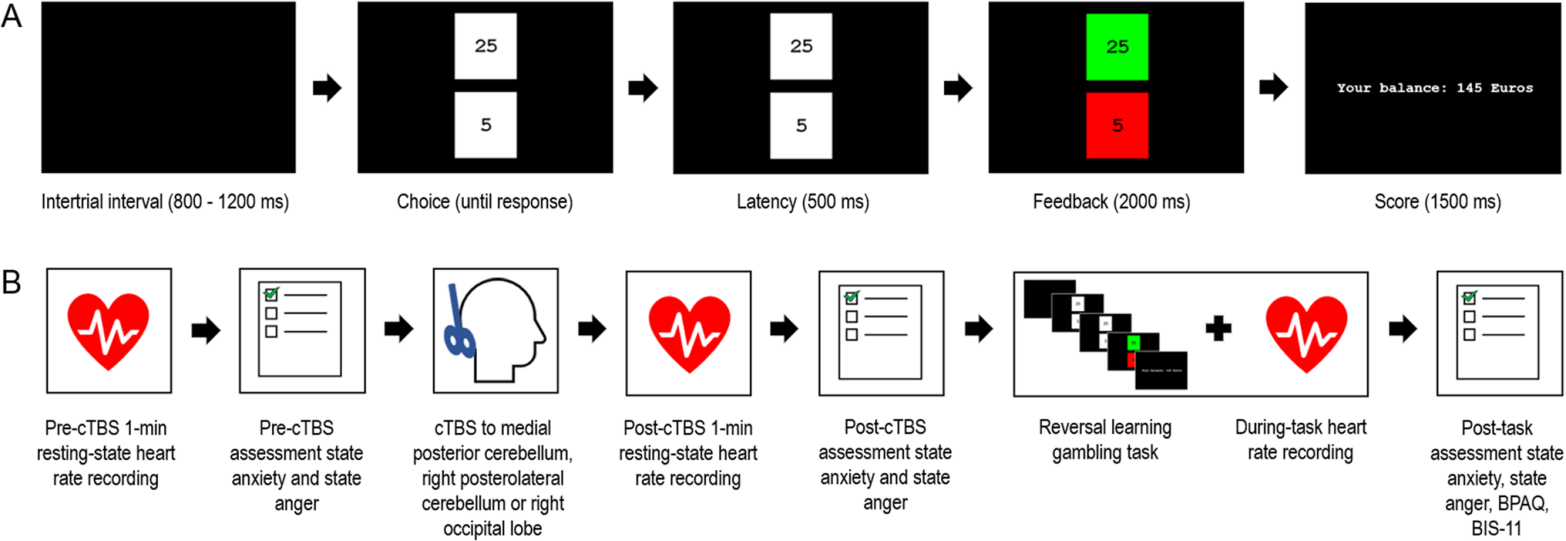
**A)** Example of a trial sequence of the reversal learning gambling task. **B)** Overview of the study procedures

The reversal learning gambling task as previously validated and described by Wischnewski and colleagues (2016; 2020) and Schutte and colleagues (2017) was used to assess reversal learning. On each trial, participants chose one of two numerical values representing fictitious money that could be won or lost. Participants could choose the higher numerical value (relatively large reward/loss for correct/incorrect choice) or the lower numerical value (relatively small reward/loss for correct/incorrect choice). The task consisted of three phases that differed in their reward-punishment contingency schedule. In phase 1 (blocks 1 and 2, trials 1–40), the higher numerical value was rewarded in 80% of the trials. In phase 2 (blocks 3 and 4, trials 41–80), the reward-punishment contingency was reversed, such that the higher numerical value was rewarded in only 20% of the trials. In phase 3 (blocks 5 and 6, trials 81–120), the reward-punishment contingency was reversed, such that the higher numerical value was again rewarded in 80% of the trials. Thus, in the first and third phase of the task, choosing the higher numerical value was the optimal strategy, whereas choosing the lower numerical value was most advantageous in the second phase of the task. Figure 1A displays the sequence of a trial.

### Continuous theta burst stimulation

Continuous theta burst stimulation was applied to the medial posterior cerebellum, right posterolateral cerebellum, or right occipital lobe using a Magstim double 70-mm figure-of-eight air film coil connected to a Magstim Rapid^2^ magnetic stimulator (Magstim, Whitland, UK). The stimulation protocol consisted of three-pulse 50-Hz bursts that were repeated every 200 ms for 40 s, amounting to 600 pulses in total (Huang et al., 2005). The coil was placed tangentially on the head with the handle pointing upwards. The medial posterior cerebellum was defined as the location 1.5 cm below the inion (EEG electrode position Iz). While the location of 1 cm below the inion is most commonly used (van Dun et al., 2017), we applied cTBS 1.5 cm below the inion with the aim to reduce the likelihood of inducing currents in the occipital lobe (Hardwick et al., 2014). The right posterolateral cerebellum was defined as the location 1 cm below and 3 cm right lateral to the inion (van Dun et al., 2017). The right occipital lobe served as an active control condition and was localized at EEG electrode position O2. Participants were randomly assigned to receive cTBS to either the medial posterior cerebellum, right posterolateral cerebellum or right occipital lobe. Randomization was stratified for age. Stimulation intensity was fixed to 45% of maximum stimulator output and was deemed safe and successful in influencing cerebellar neuronal activity in neurotypical volunteers (Hurtado-Puerto et al., 2020). Stimulation parameters were in accordance with the safety guidelines of the International Federation of Clinical Neurophysiology (Rossi et al., 2021).

### Heart Rate Variability

Electrocardiography was performed using a BioSemi ActiveTwo system (BioSemi, Amsterdam, The Netherlands). Electric signals from the heart were detected by three Ag/AgCl electrodes filled with conductive electrode gel. The first active electrode was placed under the right collarbone, the second active electrode was placed under the lowest left rib and a ground electrode was placed on the right abdomen (Kruithof et al., 2024).

### Affective State-trait Factors

The state anxiety scale of the State-Trait Anxiety Inventory (STAI; Spielberger et al., 1970) was used to assess state anxiety based on 20 items, and the state anger scale of the State-Trait Anger Expression Inventory-2 (STAXI-2; Spielberger, 1999) was used to assess state anger based on ten items. For both state scales, participants responded to the items on a four-point scale ranging from 1 (not at all) to 4 (very much so). The Buss-Perry Aggression Questionnaire (BPAQ; Buss & Perry, 1992) assessed trait aggression based on 29 items to which participants responded on a five-point scale ranging from 1 (extremely uncharacteristic of me) to 5 (extremely characteristic of me). The Barratt Impulsiveness Scale (BIS-11; Patton et al., 1995) assessed trait impulsivity based on 30 items to which participants responded on a four-point scale ranging from 1 (rarely/never) to 4 (almost always/always). A higher score on the questionnaires corresponded to a higher level of the respective state/trait.

## Procedure

Volunteers were told that the aim of the study was to investigate the role of the cerebellum in decision-making. In the days before the session, participants received the study information letter and the cTBS screening form. Participants were requested to refrain from caffeine-containing drinks and chocolate 2 h before the test session and alcohol 24 h in advance. At the start of the session, each participant filled out the screening form to check for contraindications of cTBS and provided informed consent. Next, the electrodes for electrocardiography were applied and heart rate was recorded in resting-state for 1 min—the minimum duration recommended to obtain reliable measures of HRV that reflect vagal tone (Laborde et al., 2017). The resting-state recording was taken while participants sat with their knees at a 90° angle, had both feet flat on the floor and eyes closed. Afterwards, participants filled out the state anxiety scale and the state anger scale. An EEG cap was placed on the participant’s head and centered at the vertex according to the international 10–10 EEG system to determine stimulation site. Participants then received cTBS to either the medial posterior cerebellum, right posterolateral cerebellum, or right occipital lobe while seated in a massage chair in upright position and laying with their head on a headrest. This was followed by another 1-min resting-state heart rate recording and assessment of state anxiety and state anger. Participants then engaged in the reversal learning gambling task, during which heart rate was recorded as well. After the task, participants filled out the state anxiety and state anger scale, the BPAQ and the BIS-11. Participants were debriefed at the end of the session. Figure 1B displays an overview of the study procedures.

## Data Reduction and Statistical Analyses

A 4-parameter logistic least-squares curve was fit to the trial-averaged probability of a higher numerical value decision for each cTBS condition (Wischnewski et al., 2016, 2020) according to the formula: *y* = ${H}_{\text{min}}+ \frac{{H}_{\text{max}} - {H}_{\text{min}}}{1+({\frac{x}{I50})}^{-Hill}}$. The parameters *I*50, *H*_min_ and *H*_max_ in this formula were of interest for the current study. The inflection point *I*50 is the midpoint between the lower and upper asymptote and indicates trial number *x* where the reversal has been learned. The* I*50 is operationalized as the cognitive component of reversal learning. The parameters *H*_min_ and *H*_max_ represent the lower and upper asymptote (minimum and maximum probability of a higher numerical value decision), respectively. *H*_min_ and *H*_max_ are measures of implementation of the optimal strategy and operationalized as the affective component of reversal learning. For the first reversal (at trial 41), averaged data from trials 21–80 was used. For the second reversal (at trial 81), averaged data from trials 61–120 was used. Note that 20 trials (i.e., trials 61–80) are present in the 4-parameter logistic curve fits for both reversals, which was done to keep the number of trials that were used the same for both reversals. To investigate differences in learning rate and the baseline level of implementation of the optimal strategy in the first task block, a linear regression was performed on averaged data from trials 1–20.

With regard to the heart rate data preprocessing, MATLAB R2023b (MathWorks) was used to identify the peaks (R peaks) in the heart rate signal. Based on these peaks, the interbeat intervals (IBIs; time differences between successive heart beats in ms) were calculated. Very short (< 500 ms) and very long (> 1500 ms) IBIs were removed, based on reports of normal IBIs during mental demands (mean + 3 *SD*) and during rest (mean − 3 *SD*) in healthy adults, respectively (Garde et al., 2002; Quer et al., 2020). The IBIs were used to retrieve the root mean square of successive differences (RMSSD) measure of HRV, which reflects vagal tone (Laborde et al., 2017), in Kubios HRV Standard version 3.5 (Tarvainen et al., 2014). Threshold artifact correction was applied to the heart rate data, which compares every IBI against a local average, and identifies an R peak as artifact if it exceeds the specified threshold. The identified artifacts were replaced by interpolated values using a cubic spline interpolation. A low artifact correction threshold was applied, as recommended for young adults (Alcantara et al., 2020). For a heart rate of 60 beats/min, this corresponds to the rejection of IBIs that are smaller/larger than 0.35 s compared with the local average. This criterion becomes smaller as heart rate increases.

For each questionnaire, after reverse-scoring the relevant items, total scores were calculated by summing the scores of the items.

Appropriateness of using the linear regression and the 4-parameter logistic function for the first task block and the two reversals, respectively, was verified by comparing the corrected Akaike information criterion (AICc) for both model fits for each cTBS condition. The linear regression (i.e., regression coefficient and intercept) and logistic regression parameters were compared between cTBS conditions using a one-way ANOVA. Significant differences were followed up with uncorrected post-hoc *t*-tests to explore group differences.

Additionally, separate one-way ANCOVAs were performed to assess whether HRV, state anxiety, state anger, trait aggression and trait impulsivity modulated the effect of cTBS condition on the implementation of the optimal strategy. Note that the 4-parameter logistic regression is performed on averaged data per cTBS condition and thus does not provide data for individual participants. Therefore, the proportion of higher numerical value decisions in each task phase was used as a measure of rule implementation in these analyses. Interactions of cTBS condition with HRV, state anxiety and state anger were tested for all assessments (i.e., pre- and post-cTBS, during-task for HRV and post-task for the state scales). For interactions between cTBS condition and during-task HRV, the RMSSD was calculated for the respective task phase. Due to technical issues, HRV data were incomplete for two participants (*n* = 1 missing post-cTBS and task phase 1 and *n* = 1 missing task phases 1 and 2). Available HRV data for these participants were included in the rest of the analyses.

Separate one-way ANOVAs were performed to examine pre-cTBS differences between cTBS conditions in HRV, state anxiety and state anger. A one-way ANOVA on the normalized pre- to post-cTBS RMSSD change was performed to investigate a possible direct or indirect effect of cerebellar cTBS on HRV by stimulation of the cervical vagal nerve. Additionally, separate one-way ANOVAs were performed to examine differences between cTBS conditions in the normalized pre- to post-cTBS change in state anxiety and state anger.

All statistical analyses were performed in R version 4.3.0 (R Core Team, 2023). The alpha level of significance was set to 0.05 (two-tailed) throughout. No correction for multiple testing was applied as this is the first non-invasive brain stimulation study to explore the contribution of the cerebellum to reversal learning and the potential mechanisms involved.

## Results

Continuous theta burst stimulation was overall well tolerated and no serious adverse events were observed. A small proportion of participants (3.6%; similar to Hurtado-Puerto et al., 2020) reported mild negative side effects which included pain in the stimulation area (*n* = 2 and *n* = 1 in the medial posterior and right posterolateral cerebellum cTBS condition, respectively) and blurry vision (*n* = 1, right occipital lobe cTBS condition). The side effects were transient and short-lasting. Pain in the stimulation area disappeared spontaneously in all participants after cTBS administration. Blurry vision following cTBS to the right occipital lobe lasted for a few minutes after termination of cTBS and the participant was able to continue the experimental procedures afterwards. Results showed no differences between cTBS conditions in pre-cTBS HRV (*F*(2,108) = 0.914, *p* = 0.404), state anxiety (*F*(2,108) = 0.785, *p* = 0.459) and state anger (*F*(2,108) = 0.336, *p* = 0.715). Furthermore, no difference was observed between cTBS conditions in the normalized pre- to post-cTBS HRV change (*F*(2,107) = 2.481, *p* = 0.088), making either direct or indirect stimulation of the cervical vagal nerve by cerebellar cTBS less likely. Additionally, there were no differences between cTBS conditions in the normalized pre- to post-cTBS change in state anxiety (*F*(2,108) = 0.614, *p* = 0.543) and state anger (*F*(2,108) = 0.346, *p* = 0.708). Table 1 shows the descriptive statistics of the sample demographics, HRV levels, questionnaire scores and proportion higher numerical value decisions for each cTBS condition.

**Table 1:** Descriptive statistics of the sample demographics, HRV levels, questionnaire scores and proportion higher numerical value decisions for each cTBS condition (presented as *M* ± *SD* for continuous variables and as total count for categorical variables)

		Medial posterior cerebellum (*n* = 37)	Right posterolateral cerebellum (*n* = 37)	Right occipital lobe (*n* = 37)
Demographics				
	Age	22.57 ± 2.57	22.57 ± 3.12	22.51 ± 2.58
	Number of males	5	7	6
HRV (in ms)				
	Pre-cTBS RMSSD	44.38 ± 22.62	48.01 ± 21.94	53.12 ± 36.61
	Post-cTBS RMSSD	53.65 ± 27.67	58.77 ± 29.48	52.85 ± 28.63
	RMSSD phase 1	47.08 ± 25.04	45.98 ± 23.12	47.86 ± 28.99
	RMSSD phase 2	46.40 ± 26.03	44.98 ± 21.84	43.86 ± 26.08
	RMSSD phase 3	43.92 ± 22.42	44.85 ± 21.54	45.23 ± 26.78
Questionnaires				
	Pre-cTBS state anxiety	31.68 ± 7.28	33.70 ± 7.11	32.57 ± 6.52
	Post-cTBS state anxiety	31.71 ± 7.71	33.67 ± 8.12	34.41 ± 9.33
	Post-task state anxiety	30.87 ± 6.26	34.35 ± 8.20	33.11 ± 7.91
	Pre-cTBS state anger	10.65 ± 1.14	10.95 ± 2.99	10.60 ± 1.26
	Post-cTBS state anger	10.57 ± 1.32	10.78 ± 1.93	10.73 ± 1.73
	Post-task state anger	10.60 ± 1.14	11.38 ± 3.13	10.81 ± 1.94
	BPAQ total score	59.03 ± 12.93	60.46 ± 12.46	58.78 ± 12.56
	BIS-11 total score	59.68 ± 9.11	59.68 ± 10.31	59.54 ± 9.55
Higher numerical value decisions				
	% Decisions phase 1	63.04 ± 14.90	68.24 ± 14.43	66.55 ± 13.36
	% Decisions phase 2	38.24 ± 15.62	35.20 ± 12.89	33.31 ± 14.85
	% Decisions phase 3	67.70 ± 18.93	75.14 ± 17.37	73.72 ± 17.65

*BPAQ* Buss-Perry Aggression Questionnaire, *BIS-11* Barratt Impulsiveness Scale, *RMSSD* root mean square of successive differences

Comparisons of the AICc confirmed that the linear regression provided the best fit for the first task block (trials 1–20), whereas the 4-parameter logistic regression was the best fit for the first and second reversal in all cTBS conditions (Supplementary Table 1).

**Fig. 2:**
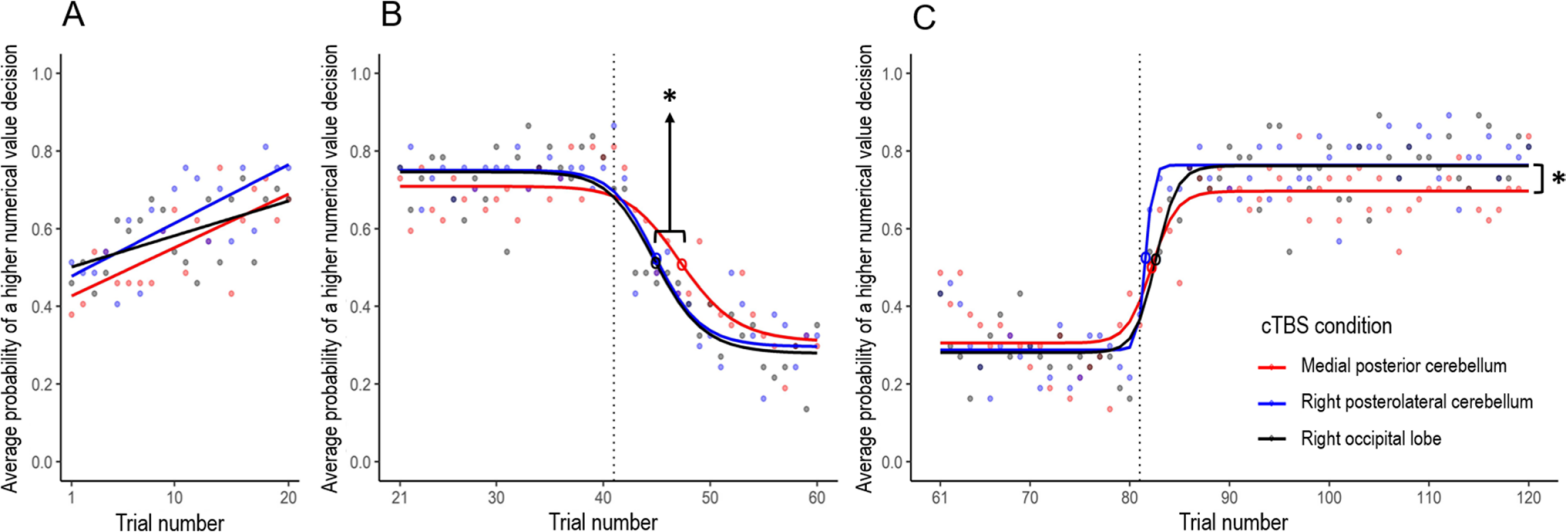
Reversal learning results for **A**) the first task block based on the linear regression, **B**) the first reversal based on the 4-parameter logistic regression, and **C**) the second reversal based on the 4-parameter logistic regression. Note that for the first reversal, data were actually fitted until trial 80 but the fitted data from trials 61–80 are not shown, because the data of these trials were also fitted for the second reversal. The first reversal (at trial 41) and second reversal (at trial 81) are indicated by a dotted line. Inflection points (parameter *I*50) are indicated by a circle. Significant differences (*p* < 0.05) are indicated by an asterisk

The linear regression of the first task block showed no differences between cTBS conditions (Table 2; Fig. 2A) in learning rate (regression coefficient; *F*(2,108) = 1.148, *p* = 0.321) nor in baseline implementation of the optimal strategy (intercept; *F*(2,108) = 1.121, *p* = 0.330).

**Table 2:** Parameter values (mean and standard error of the mean) for each cTBS condition for the first task block (based on the linear regression) and the first reversal and second reversal (both based on the 4-parameter logistic regression)

		Medial posterior cerebellum (*M* ± *SEM*)	Right posterolateral cerebellum (*M* ± *SEM*)	Right occipital lobe (*M* ± *SEM*)
First task block				
	Coefficient	0.014 ± 0.003	0.015 ± 0.003	0.009 ± 0.003
	Intercept	0.413 ± 0.037	0.463 ± 0.039	0.493 ± 0.037
Reversal 1				
	*H*_min_	0.308 ± 0.017	0.296 ± 0.015	0.279 ± 0.015
	*H*_max_	0.709 ± 0.019	0.751 ± 0.018	0.746 ± 0.018
	*I*50	47.350 ± 0.905	44.957 ± 0.664	44.901 ± 0.675
Reversal 2				
	*H*_min_	0.306 ± 0.019	0.288 ± 0.017	0.281 ± 0.018
	*H*_max_	0.697 ± 0.014	0.764 ± 0.012	0.762 ± 0.013
	*I*50	82.187 ± 0.598	81.585 ± 0.251	82.613 ± 0.433

After the first reversal, a significant difference in the *I*50 parameter was found between cTBS conditions (*F*(2,108) = 3.325, *p* = 0.040; Table 2; Fig. 2B). Post-hoc *t*-tests revealed a higher *I*50, indicating slower reversal learning in the medial posterior cerebellum compared to the right posterolateral cerebellum (*t*(72) = 2.132, *p* = 0.036) and right occipital lobe cTBS condition (*t*(72) = 2.169, *p* = 0.033). The *I*50 parameter did not differ between the right posterolateral cerebellum and right occipital lobe cTBS condition (*t*(72) = 0.059, *p* = 0.953). No differences in the *H*_min_ (*F*(2,108) = 0.844, *p* = 0.433) and *H*_max_ (*F*(2,108) = 1.510, *p* = 0.226) parameter were found between cTBS conditions for the first reversal (Table 2; Fig. 2B).

After the second reversal, a significant difference in *H*_max_ was observed between cTBS conditions (*F*(2,108) = 8.349, *p* < 0.001; Table 2; Fig. 2C). Post-hoc *t*-tests showed reduced implementation of the optimal strategy as indicated by a lower probability to choose the higher numerical value in the medial posterior cerebellum compared with the right posterolateral cerebellum (*t*(72) =  − 3.637, *p* < 0.001) and right occipital lobe cTBS condition (*t*(72) =  − 3.407, *p* = 0.001). The *H*_max_ parameter did not differ between the right posterolateral cerebellum and right occipital lobe cTBS condition (*t*(72) = 0.113, *p* = 0.910). No differences in the *I*50 (*F*(2,108) = 1.280, *p* = 0.282) and *H*_min_ (*F*(2,108) = 0.497, *p* = 0.610) parameter were found between cTBS conditions for the second reversal (Table 2; Fig. 2C).

No interactions of cTBS condition with HRV, state anxiety, state anger, trait aggression, and trait impulsivity on the proportion higher numerical value decisions in each task phase were observed (see Supplementary Table 2 for details).

## Discussion

The aim of this study was to examine the direct contribution of the cerebellum to reward- and punishment-based reversal learning. Contrary to our hypothesis, cTBS to the medial posterior, but not right posterolateral cerebellum reduced reversal learning rate, and only after the first reversal. Additionally, in line with our hypothesis, results showed that cTBS to the medial posterior cerebellum diminished the implementation of the optimal strategy, but only after the second reversal had been learned.

Slower learning after the first reversal following cTBS to the medial posterior cerebellum may be explained by its role in processing reward and punishment consequences of actions (Kobza & Bellebaum, 2015; Kruithof et al., 2023; Larry et al., 2024; Späti et al., 2014). For example, the posterior vermis processes performance-dependent reward and punishment feedback (Kruithof et al., 2023; Späti et al., 2014). Action-dependent feedback processing in the posterior vermis was also found by Kobza and Bellebaum (2015), who reported activity in this region during the processing of reward prediction errors for own compared with observed actions of others (Kobza & Bellebaum, 2015). Furthermore, posterior vermis neurons code received and omitted reward outcomes of actions in monkeys (Larry et al., 2024). Processing reward and punishment consequences of actions is arguably necessary to signal the need to update the internal model to adjust behavior in response to changes in reward-punishment contingencies (Blackwood et al., 2004; Kostadinov & Häusser, 2022; Kruithof et al., 2023; Peterburs et al., 2018). Thus, cTBS to the medial posterior cerebellum may have interfered with processing action-related reward and punishment feedback and updating of the internal model. This interference may reflect increased uncertainty due to a more noisy system caused by cTBS as a result from disruptions in signal transfer between the cerebellum and basal ganglia (Kostadinov & Häusser, 2022; Larry et al., 2024). More specifically, cTBS to the medial posterior cerebellum may have hampered the use of reward signals received from the basal ganglia to learn the value of actions necessary to update action-outcome predictions (Kostadinov & Häusser, 2022; Schultz et al., 1998). Evidence has shown that the medial posterior cerebellum is particularly involved in predicting immediate rewards, while the left posterolateral cerebellum predicts rewards of future states (Tanaka et al., 2004). This observation is in line with the idea that the medial cerebellum is implicated in model-free reinforcement learning (Terburg et al., 2024). That is, values of actions are learned through trial-and-error instead of by forming feedforward predictions about action consequences based on an internal model (Drummond & Niv, 2020). While in conflict with the view that the medial posterior cerebellum processes reward and punishment signals to update internal models, our results suggest that the current task predominantly tapped into a model-free learning strategy. Indeed, reversal learning in the current task could be successful by adopting a win-stay, lose-shift strategy (Izquierdo et al., 2017). Conversely, a model-based learning strategy may engage more posterolateral cerebellar regions (Huo et al., 2023; Terburg et al., 2024). However, the presently used task does not allow us to dissociate model-free and model-based strategies. Whether the medial posterior cerebellum is more involved in model-free learning remains to be empirically tested in future research. Interestingly, slower learning was no longer observed after the second reversal. This suggests that learning the first reversal depends more on processing reward and punishment consequences of actions, whereas learning the second reversal may involve some sort of meta-learning rooted in the participant’s knowledge about the first reversal (Wischnewski et al., 2016). As such, meta-learning relies to a lesser extent on involvement of the medial posterior cerebellum. Indeed, a post-hoc analysis showed that the second reversal was learned faster than the first reversal across all cTBS conditions (*p*s < 0.001), supporting the notion that learning the second reversal involves meta-learning.

Diminished implementation of the optimal strategy after learning the second reversal (Wischnewski et al., 2016) following cTBS to the medial posterior cerebellum may be explained in terms of its role in the propensity to accommodate immediate rewards (Lai et al., 2024; Manto et al., 2024; Tanaka et al., 2004). Continuous theta burst stimulation to the medial posterior cerebellum may have gradually reduced reward seeking behavior (Fecteau et al., 2007), thereby decreasing selection of the response option associated with the highest reward probability. The diminished implementation of the optimal strategy after learning the second reversal may perhaps depend on interactions with the basal ganglia. That is, cTBS to the medial posterior cerebellum may have interfered with the transmission of action-outcome predictive signals toward the basal ganglia to reinforce actions that will lead to reward (Kostadinov & Häusser, 2022). Speculatively, the suboptimal implementation of the learned strategy may suggest less exploitation of learned response options with predictive reward outcomes in favor of exploration of potentially more profitable but riskier response options. Indeed, exploitation of acquired knowledge is the optimal strategy after a change in reward-punishment contingency has been learned (Cohen et al., 2007; Navarro et al., 2016). The implementation of the optimal strategy before (i.e., choosing the higher numerical value) and after (i.e., choosing the lower numerical value) learning the first reversal was unaffected following medial posterior cerebellar cTBS. This may potentially be explained by a shift in the exploration–exploitation trade-off that developed throughout the task in the medial posterior cerebellum cTBS condition. Specifically, the repeated encounters with changes in reward-punishment contingencies may have induced uncertainty and gradually increased the urge to adopt an exploratory strategy towards the end of the task (Cohen et al., 2007; Navarro et al., 2016). This unfavorable uncertainty-induced exploratory strategy may have been instigated in the earlier mentioned interaction with the basal ganglia, which controls the exploration–exploitation tradeoff as a function of uncertainty (Gilbertson & Steele, 2021; Humphries et al., 2012). Alternatively, cTBS to the medial posterior cerebellum may have gradually resulted in the use of a more conservative strategy, characterized by choosing the response option that is associated with the lowest likelihood of receiving a relatively large loss.

The absent effect of right posterolateral cerebellar cTBS on reversal learning in the current study is in contrast with evidence of impaired reversal learning in mice following chemogenetic inhibition of Purkinje cells in right Crus I (Stoodley et al., 2017). Moreover, the absent effect appears not to be in line with a recent review that showed involvement of bilateral Crus I and II in feedback learning in a non-motor context (Berlijn et al., 2024). Given the role of the posterolateral cerebellum in higher-order cognitive functions (Keren-Happuch et al., 2014; King et al., 2019; Stoodley & Schmahmann, 2009), the null effect suggests that the task used in the current study relies less on cognitive processes involved in reversal learning. With regard to the evidence that learning the second reversal involved meta-learning, which does rely on higher-order cognitive processes, reduced meta-learning after the second reversal may have been expected following cTBS to the right posterolateral cerebellum. The absent effect of right posterolateral cerebellar cTBS on reversal learning may stem from neural compensation mechanisms (Mitoma et al., 2020). Cerebellar lesion studies in humans (Adamaszek et al., 2013; Pereira et al., 2017) and non-human primates (Mackel, 1987) have found evidence for compensation of motor, cognitive, and affective processes by extracerebellar regions. A candidate region for neural compensation may be the prefrontal cortex given its anatomical (Palesi et al., 2015) and functional (Buckner et al., 2011; Sang et al., 2012) connections to the posterolateral cerebellum and its well-documented role in reversal learning (Yaple & Yu, 2019). Another possibility is that cerebellar regions outside of the targeted area may have compensated for the functional loss of the perturbed region (Mitoma et al., 2020, 2021). The left posterolateral cerebellum may, for example, have offset the reduced activity of the right homologue (Verpeut et al., 2023). Future research could test this theory by applying cTBS to the left and right posterolateral cerebellum simultaneously and comparing reversal learning performance of those individuals to the performance of individuals who received cTBS to only one hemisphere. Alternatively, the left posterolateral cerebellum may even be more important than the right posterolateral cerebellum in reversal learning (Peterburs et al., 2018). Peterburs et al. (2018) reported activity in the left posterolateral cerebellum of healthy adults associated with the initiation of a change in response strategy on a reversal learning task which was more complex and cognitively demanding than our task. Consequently, the task used by Peterburs et al. (2018) may have more readily engaged the posterolateral cerebellum. Future research is needed to examine the effect of cerebellar non-invasive brain stimulation on reversal learning with increased cognitive demand. In combination with increased cognitive load, the left posterolateral cerebellum may be involved in a reversal learning task which starts and ends with a phase where the focus is on avoiding relatively high punishments as opposed to obtaining relatively high rewards. This idea is based on the cerebellar lateralization hypothesis of motivational direction that attributes a role of the left posterolateral cerebellum to punishment-related motivational processes (Kruithof et al., 2022, 2024, 2025; Schutter, 2020).

Continuous theta burst stimulation may have influenced tissue in close proximity to the region of interest (Bijsterbosch et al., 2012; Janssen et al., 2014; Klaus, & Schutter, 2022). In particular, cerebellar cTBS may have induced electric currents in the inferior part of the occipital lobe in some individuals (Bijsterbosch et al., 2012; Hardwick et al., 2014; Janssen et al., 2014; Klaus & Schutter, 2022). To control for this possibility, right occipital lobe cTBS was included as an active control condition. Additionally, task performance in the right occipital lobe cTBS condition is comparable (Supplementary Fig. 1) to task performance observed in a previous EEG study in healthy volunteers (Schutte et al., 2017). Therefore, it seems less likely that task performance in the medial posterior cerebellum cTBS condition can be attributed to occipital lobe perturbation. However, since task performance in the right posterolateral cerebellar cTBS condition resembles task performance in the right occipital lobe cTBS condition, we cannot exclude the possibility that the absent effects in the right posterolateral cerebellar cTBS condition are due to occipital lobe perturbation. Moreover, the effect of medial posterior cerebellar cTBS on reversal learning may (partly) be due to perturbation of either the left or both left and right posterolateral cerebellum. Particularly because cTBS was not applied to the left posterolateral cerebellum, we cannot draw definitive inferences regarding the functional specificity of the medial posterior and right posterolateral cerebellum in reversal learning. Future research is needed to further disentangle the contribution of different regions of the cerebellum to reversal learning and the underlying processes they subserve.

In line with our findings, cerebellar ataxia is associated with impairments in reversal learning and implementation of the optimal behavioral strategy (Nicholas et al., 2024; Thoma et al., 2008). Our findings may contribute to insights on potential target locations for non-invasive brain stimulation treatment of patients with cerebellar lesions, as well as for disorders characterized by mental inflexibility, such as addiction (Ngetich et al., 2024), psychopathy (Budhani et al., 2006; Dargis et al., 2017), and obsessive-compulsive disorder (Tezcan et al., 2017).

In contrast to our recent study that reported associations of state anger and heart rate with aggressive behavior during cerebellar transcranial direct current stimulation (Kruithof et al., 2024), no evidence was found for interactions of cerebellar cTBS with HRV and affective states and traits in the current study. A limited range in affective state and trait scores across our unselected healthy participants together with a weak or absent association of HRV and the state and trait scores with task performance may explain these null findings.

Several limitations of the study should be addressed. First, because the results were not corrected for multiple testing, the significant finding of slower learning after the first reversal following cTBS to the medial posterior cerebellum should be interpreted with caution. Second, the potential interindividual variability of cTBS-related effects in targeted cerebellar areas should be considered (Klaus & Schutter, 2022). Given the high level of cortical folding in the cerebellum, relatively small interindividual anatomical variation can have a substantial influence on brain stimulation responses in the cerebellum compared to other brain regions (Darch et al., 2018; Ponce et al., 2022). The use of fiducial landmarks in the current study to determine stimulation site likely contributed to interindividual variability in targeted regions (Sack et al., 2009). Neuronavigated TMS based on individual structural MRI scans could increase the accuracy of targeting the region of interest (Herwig et al., 2001). Additionally, electric field simulations based on MRI scans can be informative with respect to the focality of the electric current induced with cTBS on the individual level. Despite these limitations, this is, to our knowledge, the first non-invasive brain stimulation study to find direct evidence for cerebellar involvement in reward- and punishment-based reversal learning in healthy volunteers.

## Conclusions

The findings suggest a role of the cerebellum in reversal learning and behavioral adaptation, possibly by regulating decision-based uncertainty in a volatile environment.

## Supplementary Information

Below is the link to the electronic supplementary material.

Supplementary file1 (DOCX 2.55 MB)

## Data Availability

The scripts used for this article are available at Yoda and can be accessed upon request at 10.24416/UU01-V10YJ8.

## Declarations

### Data availability and open practices statement

The data used for this article are available at Yoda and can be accessed upon request at 10.24416/UU01-V10YJ8. The study was not preregistered.
