# Supplementary material for: Continuous theta burst stimulation to the medial posterior cerebellum impairs reversal learning in healthy volunteers
Source: Cogn Affect Behav Neurosci. 2025 Mar 26;25(3):618–30. doi: 10.3758/s13415-025-01273-5 (PMC12130073; doi:10.3758/s13415-025-01273-5)
Supplement: Supplementary file 1 — Supplementary file1 (DOCX 2.55 MB) [file 13415_2025_1273_MOESM1_ESM.docx]

**Supplementary materials**

**Table 1.** Comparisons of the corrected Akaike information criterion between the linear and 4-parameter logistic model for the first task block and the first and second reversal for each cTBS condition. Note that lower values indicate a better model fit.

|  | Medial posterior cerebellum | Right posterolateral cerebellum | Right occipital lobe |
| --- | --- | --- | --- |
| *First task block (trials 1-20)* |  |  |  |
| Linear model | -38.94 | -36.92 | -38.66 |
| 4-parameter logistic model | -32.29 | -32.21 | -32.51 |
|  |  |  |  |
| *Reversal 1 (trials 21-80)* |  |  |  |
| Linear model | -97.08 | -92.76 | -84.73 |
| 4-parameter logistic model | -125.75 | -132.54 | -133.05 |
|  |  |  |  |
| *Reversal 2 (trials 61-120)* |  |  |  |
| Linear model | -70.74 | -65.61 | -68.73 |
| 4-parameter logistic model | -125.97 | -135.69 | -129.29 |

**Table 2.** Statistical results of the interactions between cTBS condition and HRV, state anxiety, state anger, trait aggression and trait impulsivity on the proportion higher numerical value decisions during phase 1-3 of the reversal learning gambling task (*N* = 111 unless indicated otherwise).

|  |  | *F* | *p* |
| --- | --- | --- | --- |
| Phase 1 |  |  |  |
|  | cTBS * pre-cTBS RMSSD | 1.748 | 0.179 |
|  | cTBS * post-cTBS RMSSD (*n* = 110) | 0.824 | 0.441 |
|  | cTBS * phase 1 RMSSD (*n* = 109) | 0.973 | 0.381 |
|  | cTBS * pre-cTBS state anxiety | 0.687 | 0.505 |
|  | cTBS * post-cTBS state anxiety | 0.245 | 0.783 |
|  | cTBS * post-task state anxiety | 0.205 | 0.815 |
|  | cTBS * pre-cTBS state anger | 0.713 | 0.493 |
|  | cTBS * post-cTBS state anger | 0.981 | 0.378 |
|  | cTBS * post-task state anger | 1.397 | 0.252 |
|  | cTBS * BPAQ total score | 1.563 | 0.214 |
|  | cTBS * BIS-11 total score | 0.175 | 0.840 |
|  |  |  |  |
| Phase 2 |  |  |  |
|  | cTBS * pre-cTBS RMSSD | 0.680 | 0.509 |
|  | cTBS * post-cTBS RMSSD (*n* = 110) | 1.319 | 0.272 |
|  | cTBS * phase 2 RMSSD (*n* = 110) | 0.738 | 0.480 |
|  | cTBS * pre-cTBS state anxiety | 0.032 | 0.968 |
|  | cTBS * post-cTBS state anxiety | 1.031 | 0.360 |
|  | cTBS * post-task state anxiety | 0.788 | 0.457 |
|  | cTBS * pre-cTBS state anger | 0.659 | 0.520 |
|  | cTBS * post-cTBS state anger | 0.259 | 0.772 |
|  | cTBS * post-task state anger | 1.083 | 0.342 |
|  | cTBS * BPAQ total score | 0.068 | 0.934 |
|  | cTBS * BIS-11 total score | 1.413 | 0.248 |
|  |  |  |  |
| Phase 3 |  |  |  |
|  | cTBS * pre-cTBS RMSSD | 0.634 | 0.533 |
|  | cTBS * post-cTBS RMSSD (*n* = 110) | 0.017 | 0.983 |
|  | cTBS * phase 3 RMSSD | 0.243 | 0.785 |
|  | cTBS * pre-cTBS state anxiety | 0.993 | 0.374 |
|  | cTBS * post-cTBS state anxiety | 0.131 | 0.878 |
|  | cTBS * post-task state anxiety | 0.583 | 0.560 |
|  | cTBS * pre-cTBS state anger | 2.856 | 0.062 |
|  | cTBS * post-cTBS state anger | 0.948 | 0.391 |
|  | cTBS * post-task state anger | 2.237 | 0.112 |
|  | cTBS * BPAQ total score | 0.980 | 0.379 |
|  | cTBS * BIS-11 total score | 1.652 | 0.197 |
| BPAQ = Buss-Perry Aggression Questionnaire, BIS-11 = Barratt Impulsiveness Scale, cTBS = continuous theta burst stimulation, RMSSD = root mean square of successive differences. | | | |

**
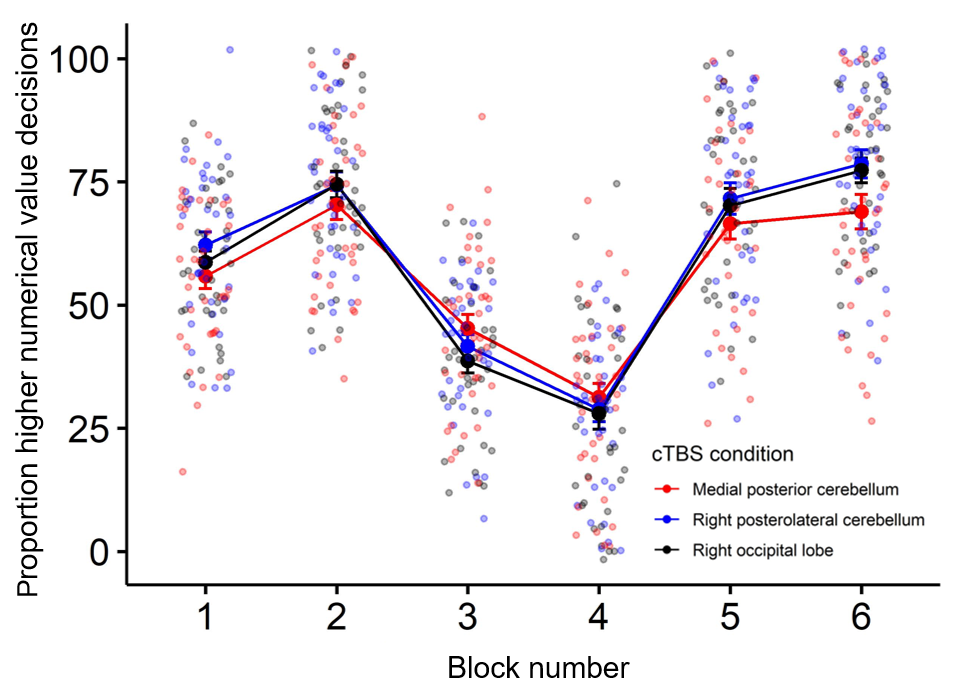
**

**Figure 1.** The proportion higher numerical value decisions in each task block for each cTBS condition. Error bars indicate standard errors of the mean. For comparison with results from an EEG study in healthy volunteers, see figure 3 in Schutte et al. (2017).
